# Duplication and Retention Biases of Essential and Non-Essential Genes Revealed by Systematic Knockdown Analyses

**DOI:** 10.1371/journal.pgen.1003330

**Published:** 2013-05-09

**Authors:** Shane Woods, Avril Coghlan, David Rivers, Tobias Warnecke, Sean J. Jeffries, Taejoon Kwon, Anthony Rogers, Laurence D. Hurst, Julie Ahringer

**Affiliations:** 1 The Gurdon Institute and Department of Genetics, University of Cambridge, Cambridge, United Kingdom; 2 Department of Microbiology, University College Cork, Cork, Ireland; 3 Wellcome Trust Sanger Institute, Wellcome Trust Genome Campus, Hinxton, Cambridge, United Kingdom; 4 Department of Biology and Biochemistry, University of Bath, Bath, Somerset, United Kingdom; California Institute of Technology, United States of America

## Abstract

When a duplicate gene has no apparent loss-of-function phenotype, it is commonly considered that the phenotype has been masked as a result of functional redundancy with the remaining paralog. This is supported by indirect evidence showing that multi-copy genes show loss-of-function phenotypes less often than single-copy genes and by direct tests of phenotype masking using select gene sets. Here we take a systematic genome-wide RNA interference approach to assess phenotype masking in paralog pairs in the *Caenorhabditis elegans* genome. Remarkably, in contrast to expectations, we find that phenotype masking makes only a minor contribution to the low knockdown phenotype rate for duplicate genes. Instead, we find that non-essential genes are highly over-represented among duplicates, leading to a low observed loss-of-function phenotype rate. We further find that duplicate pairs derived from essential and non-essential genes have contrasting evolutionary dynamics: whereas non-essential genes are both more often successfully duplicated (fixed) and lost, essential genes are less often duplicated but upon successful duplication are maintained over longer periods. We expect the fundamental evolutionary duplication dynamics presented here to be broadly applicable.

## Author Summary

Duplicate genes occur in all organisms. It has been found that mutations in duplicate genes cause defects much less often than when single copy genes are mutated. It is widely believed that this is due to functional redundancy—that is, the two genes can carry out similar functions so that the non-mutated duplicate gene can cover for or “mask” the phenotype of the mutation in the first duplicate. To determine whether this hypothesis is true, it is necessary to test systematically whether defects indeed occur in the organism when both duplicate genes are inhibited. We have for the first time carried out such an analysis in a multicellular organism, the nematode *Caenorhabditis elegans*. In contrast to expectations, we observed that when both copies of duplicate genes are inhibited deleterious effects are very rare. We show that this is because duplicate genes are much more often non-essential compared to genes where there is only a single copy. Non-essential genes are also lost from the genome much more often than essential genes. However, when essential genes are duplicated, they remain present in the genome over longer periods. Our results give a framework to explain the evolutionary dynamics of duplications in the genome.

## Introduction

**Figure 1:**
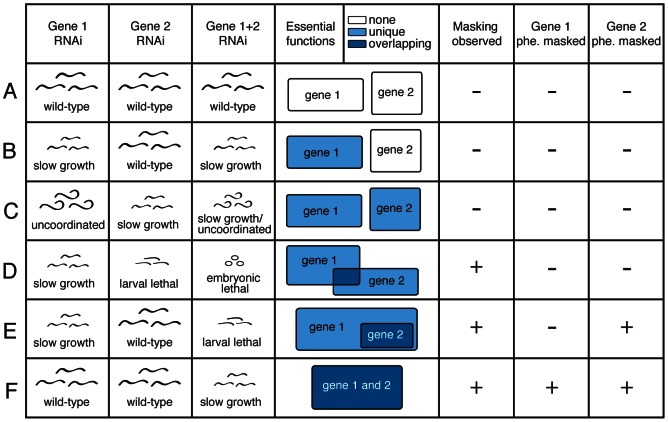
Definitions of partial and full phenotype masking. Rows represent theoretical knockdown results for duplicate pairs (A–F). The first three columns show examples of observed phenotypes for knockdown of gene 1, gene 2 or the double knockdown of both genes. Phenotype masking is scored positive if the double knockdown displays a more severe phenotype than expected under a multiplicative model of interaction, when compared to the two single-gene knockdowns (D–F; see Methods). A gene's phenotype is considered ‘fully masked’ if no observable defect is found upon single-gene knockdown, but phenotype masking is revealed upon double gene knockdown (E–F). Phenotype masking is presumed to stem from some overlap in the biochemical functions of the genes: this is shown in column 4, in which boxes represent essential gene functions defined as any apparent phenotypic defect, white indicates that we infer no essential function, light blue that we infer an essential function unique to one gene, and dark blue that we infer overlapping essential function between genes.

Duplication of genes is an important source of evolutionary novelty [1], [2]. Duplicate genes may also provide stability to an individual organism, by buffering the effect of harmful mutations [3]–[9], although it is unlikely that this explains why a duplication is initially favoured [10]. Immediately after duplication two new paralogs are probably similar in both sequence and expression. As a consequence, it is hypothesized that the effects of mutations in one paralog can be masked by the other: although the first paralog has a mutation that would normally (in the absence of masking) reduce fitness, the second paralog compensates for the mutation, so that the reduction in fitness is less than expected. This was proposed by Haldane, who hypothesised that paralogous genes could undergo mutations without disadvantage to the organism [4]. This phenomenon has been variously termed masking, functional redundancy, compensation, or phenotype buffering; we will refer to it as masking. Masking is proposed to occur because of overlap in the biochemical and physiological functions of the paralogs, which allows the second paralog to carry out the functions of the first (Figure 1).

In *Caenorhabditis elegans*, 17.7% of single-copy genes have been observed to have an ‘essential’ function, defined as a phenotypic defect easily observable upon knockdown under laboratory growth conditions [11]. Compared to single-copy genes, paralogous genes in yeast (*Saccharomyces cerevisiae*), worm (*C. elegans*), fly (*Drosophila melanogaster*) and mouse (*Mus musculus*) are significantly less likely to have a loss-of-function phenotype [11]–[16]. The low loss-of-function phenotype rates have been interpreted as evidence for functional redundancy, leading to masking of phenotypes. An alternative proposal is that duplicate genes may be biased to have originated from non-essential ancestors and that this may contribute to the lower loss of function phenotype rate of duplicate genes [17]. Phenotype masking however, remains the prevailing theory to explain why genes with paralogs more rarely have obvious loss of function phenotypes, because it is supported by relatively high observed masking rates in tests where selected samples of yeast and worm duplicate pairs have been simultaneously inhibited (∼12–55%) [18]–[22]. However, this question is still open because the incidence of masking has not yet been investigated genome-wide.

Here we report the first unbiased study of masking of duplicate gene-pairs lacking any other close homolog in a multicellular eukaryote, *C. elegans*. We observe phenotypic masking in only 6% (50/790) of duplicate gene-pairs, far less often than observed in studies of selected gene sets. Strikingly, there is an age-related bias in masking rates with younger paralog pairs (which duplicated after the *C. elegans-C. briggsae* speciation) displaying masking 4.9 times less frequently than older pairs (which arose before this speciation). We demonstrate that this rate difference is due to a large over-representation of non-essential gene pairs among younger duplicates. When considering only duplicates for which the double knockdown has a phenotype, masking rates are highest for the youngest duplicates, as expected. Our findings support a model whereby non-essential genes are both more likely to be successfully duplicated (duplicated and subsequently fixed in the population) and to be lost in the long term. However, when fixed, essential duplicates are more likely to be maintained in the long term. Overall, these evolutionary dynamics lead to a low observed loss of function phenotype rate upon knockdown of duplicate genes either singly or in pairs because they are frequently non-essential. The results indicate that phenotype masking should not be the default explanation as to why genes that have a paralog do not exhibit a discernable phenotype on single gene knockdown; it is more likely that they were derived from non-essential genes, this being especially true if they are recently duplicated.

## Results

### Only 6% of *C. elegans* paralog pairs exhibit masking

To measure the incidence of masking among paralogous genes in an unbiased way and on a genome-wide scale, we carried out single and double gene RNA interference (RNAi) knockdown experiments for 790 *C. elegans* paralog pairs (see Methods). As RNAi is a sequence-based process, a single RNAi probe will knock down both members of a pair of paralogs that have nearly identical sequences, preventing assessment of single-gene knockdown phenotypes. Therefore, a paralog pair was only included in the set of 790 pairs if they had diverged sufficiently so that a different RNAi probe could uniquely target each gene (see Methods). For each pair, the two genes are each other's closest homolog within *C. elegans* and lack any closely related paralog, although pairs may belong to a larger *C. elegans* gene family (see Methods).

To test for masking between two genes, we used the standard procedure of comparing the phenotype of each single-gene inhibition to that of the double [23]. If *w* is fitness and *s*_1_ and *s*_2_ are the reductions in fitness associated with inhibiting genes 1 and 2, then, in the absence of masking, the fitness of the single and double loss of function individuals is expected to be *w*_i_ = 1−*s*_i_, *w*_2_ = 1−*s*_2,_ and *w*_1,2_ = (1−*s*_1_)(1−*s*_2_), respectively. Fitness *w*_1,2_ lower than expected is interpreted as evidence of masking [18],[19],[24]. In some cases, both single-gene and double-gene inhibitions have no observable, or very little, reduction in fitness, (*w*_1_≈*w*_2_≈*w*_1,2_≈1), presumably because the genes are of relatively low importance to the organism in the conditions studied. Typically, genes or gene pairs where an obvious defect is observed upon knockdown (*w*_i_<1) are classified as ‘essential’ and those where no obvious phenotypic defect is observed upon knockdown (*w*_i_≈1) ‘non-essential.’ This definition of ‘essential’ genes includes those that may not have a lethal knockdown phenotype, and ‘non-essential’ genes might display a loss of function phenotype under other assay conditions or only require a very low level of gene activity to maintain fitness. In addition, classification as non-essential does not mean that the gene is evolutionarily dispensable.

Single and double RNAi knockdown experiments were conducted in duplicate using the RNAi hypersensitive strain *eri-1(mg366);lin-15B(n744)* [25]–[27]. P0s were scored for fertility and lethality of F1 embryos; P0s and F1s were additionally scored for a host of other post-embryonic phenotypes, and all observed phenotypes were confirmed by rigorous analysis of additional replicates (see Methods).

Of the genes having a single-gene knockdown phenotype in any of four previous RNAi screens [11], (*n* = 198 genes), our screen detected a single-gene knockdown phenotype in 90% of cases (Table S6). This level of concordance is similar to that observed for replicate genome-wide RNAi screens in *C. elegans* [29]. We further observed that each of the individual genes were effectively inhibited using the double RNAi feeding protocol: a phenotype was observed for 99% of double knockdowns where either of the single-gene knockdowns showed a phenotype (*n* = 175).

As described above, we considered a paralog pair to exhibit masking if the double knockdown displayed a more severe phenotype than expected under a multiplicative model of interaction when compared to the two single-gene knockdowns, *i.e. w*_1,2_<(1−*s*_1_)(1−*s*_2_) (see Methods). This includes both full and partial masking, where one member of a paralog pair either fully or partially compensates when the other member is knocked down.

We observed phenotype masking for just 6.3% (50 of 790) of paralog pairs. Surprisingly, we found that phenotype masking was very rare for genes showing no phenotypic defect upon single knockdown (5.1%, *n* = 1382). Instead, duplicate genes with single knockdown phenotypes much more often showed masking (15.2%, *n* = 198). Overall, 30% of genes displaying masking showed a single knockdown phenotype compared to 17.7% of single copy genes and 12.5% of duplicate genes.

### Masking is 4.9 times less common for younger paralog pairs than for older pairs

It is expected that masking would be more common in younger duplicates, since they generally are more similar to each other in sequence and expression [8], [13], [16], [18], [24], [31]. To investigate this we used phylogenetic analysis to identify duplicate pairs which arose from a duplication that occurred in (i) the *C. elegans* lineage after the speciation separating *C. elegans* from *C. briggsae* ∼30 Mya [32]; (ii) the ancestor of Caenorhabditis species; (iii) the ancestor of Bilateria; or (iv) the ancestor of eukaryotes (see Methods). We identified 178 duplicate pairs where the duplication occurred in the *C. elegans* lineage after speciation from *C. briggsae*, and 533 pairs that arose before this speciation (see Methods). We will refer to the 178 *C. elegans*-lineage pairs as ‘younger’ pairs, and to the 533 pairs that arose before the *C. elegans-C. briggsae* speciation as ‘older’ pairs.

Despite the expectation that masking would be most common for younger paralog pairs, we found that just 1.7% of the younger pairs (3/178 pairs) exhibited masking and 1.7% of genes in this set (6/356) exhibited a fully masked phenotype. The single-gene knockdown phenotype rate for the 356 genes in the 178 younger duplicate pairs is 1.4%, far lower than the rate of 17.7% for single-copy *C. elegans* genes (455 of 2566 genes, *X* ^2^-test: *P*<10^−14^). This 16.3% difference cannot be due to phenotypic masking since full masking is very rare among younger duplicate genes (1.7%).

**Figure 2:**
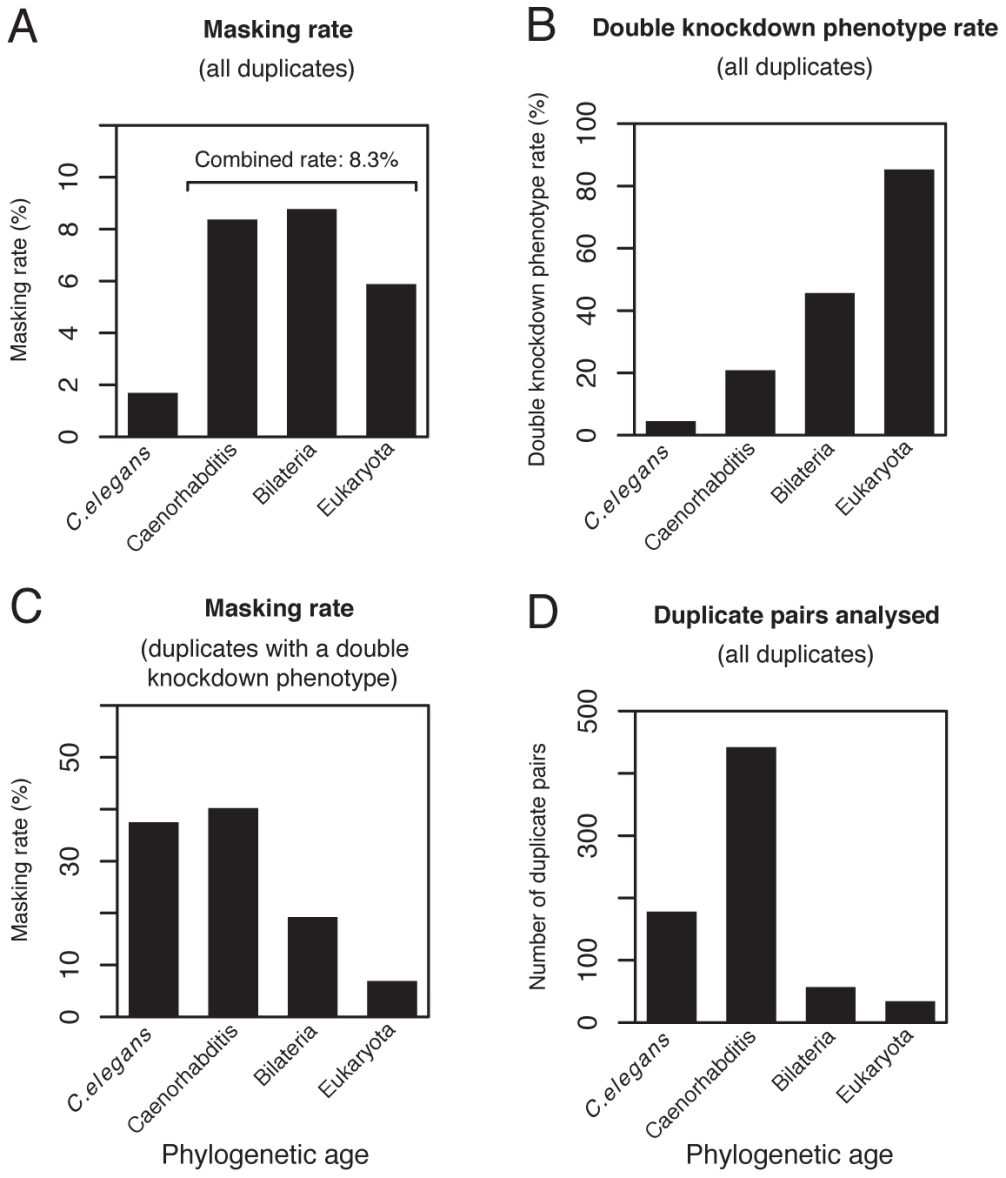
Phenotype masking and double knockdown phenotype rates grouped by phylogenetic age. (A) Masking rates (*i.e.* where the phenotype of the double knockdown was more severe than expected under a multiplicative model of interaction; this includes full and partial masking) for the subset of the 790 duplicate pairs (without a close third paralog) for which phylogenetic age could be estimated (*n* = 711 pairs for whole set; *C. elegans n* = 178; Caenorhabditis *n* = 442; Bilateria *n* = 57; Eukaryota *n* = 34). (B) Double knockdown phenotype rate for duplicate pairs in (A). (C) Masking rates for duplicate pairs in (A) considering only duplicates with a double knockdown phenotype (*n* = 155 pairs for whole set; *C. elegans n* = 8; Caenorhabditis *n* = 92; Bilateria *n* = 26; Eukaryota *n* = 29). Masking rates differ according to phylogenetic age (Fisher's test: *P* = 0.002), with a prevalence of masking amongst younger duplicate pairs. (D) Number of pairs analysed for duplicate pairs in (A).

Since a pair of duplicates will diverge over time, we would predict a lower rate of masking amongst older duplicate pairs than for younger pairs. However, surprisingly we find that overall (full or partial) and full masking rates are much higher for the 533 older pairs than the 178 younger pairs (4.9-fold and 3.4-fold, respectively Figure 2A and Figure S2).

### Masking is rare for younger paralog pairs because non-essential genes are over-represented among younger pairs

We consider a paralog pair to exhibit masking if the double knockdown displays a more severe phenotype than expected compared to the single-gene knockdown phenotypes. If a gene-pair was relatively unimportant (i.e. non-essential) under the conditions studied, then there would be no obvious phenotypic defect upon single or double knockdown and masking would not be observed. Therefore, a possible reason why masking is observed less frequently for younger than older duplicate pairs could be that a greater fraction of the younger pairs are non-essential.

If we assume that the younger duplicates have not gained or lost essential functions since the duplication events that generated them, then the extant *C. elegans* genome should be a good surrogate for the gene pool from which the duplicates arose. If so, we would predict that the fraction of younger paralog pairs that are ‘essential’ pairs (for which the double knockdown has an obvious phenotypic defect) should be approximately equal to the fraction of all *C. elegans* genes that have a single-gene knockdown phenotype. In striking contrast to this prediction, the double knockdown phenotype (essentiality) rate for the 178 younger pairs is only 4.5%, compared to 13.4% for single-gene knockdowns across the *C. elegans* genome (1917 of 14327, *X* ^2^-test: *P*<10^−3^; Figure 2B). On the other hand, the essentiality rate for the 533 older duplicate pairs is 27.6%, significantly higher than the single-gene knockdown rate for the whole *C. elegans* gene set (*X* ^2^-test: *P*<10^−15^; Figure 2B). The finding that non-essential genes are over-represented among the younger paralog pairs relative to the whole *C. elegans* gene set can explain why the observed rate of masking is low among younger paralogs: they tend to be non-essential, so display no evident phenotype upon single or double knockdown.

### Non-essential genes are more likely to be successfully duplicated than essential genes

Why are younger duplicate pairs more often non-essential compared to the whole *C. elegans* gene set (4.5% vs. 13.4%)? The young duplicate genes do not appear to be biased for particular functional classes that could explain this difference (Table S1). We also considered the possibility of masking by more distant paralogs. However, the essentiality rate for duplicate pairs with no detectable other paralog is still lower than the knockdown phenotype rate for single copy genes (Figure S1). An alternative explanation is that non-essential genes may be more likely to successfully duplicate (*i.e.* duplicate and subsequently become fixed in the population) compared to essential genes, as hypothesised by He and Zhang [17]. They showed that single-copy *S. cerevisiae* genes whose orthologs had duplicated in another yeast species were more often non-essential than those whose orthologs remained single-copy [17]. Bias favouring successful duplication of non-essential genes could explain why knockdown of duplicate pairs rarely show loss of function phenotypes. Different mechanisms could contribution to such a bias. For example, genes that are not dose sensitive on knockdown may be more prone to duplication because changes in dose are of lesser phenotypic impact.

To explore a possible duplication bias, we compared the knockdown phenotype rate of 960 *C. elegans* single-copy genes whose orthologs have remained single-copy in two other nematode species (*C. briggsae* and *C. remanei*) to that of 269 single-copy *C. elegans* genes whose orthologs have duplicated in at least one of these nematode species (see Methods). We found that the single-copy *C. elegans* genes whose orthologs have duplicated have a significantly lower knockdown phenotype rate than those whose orthologs have remained single-copy (19.3% vs. 30.2%, *X* ^2^-test: *P* = 0.0006). This agrees with a similar trend previously observed for a small *C. elegans* dataset [17]. Therefore, non-essential genes in Caenorhabditis duplicate more often than essential genes, which can explain why *C. elegans* paralog pairs are so often non-essential.

### Duplicate essential genes are more likely to be retained in the long term than non-essential duplicate genes

It is often the case that genes with an essential phenotype are more likely to have orthologs in distant species than do genes lacking any strong knockdown or knockout phenotype. Does the same hold for gene duplicates whose double knockdowns are essential or non-essential? That the duplicates with a phenotype tend to be evolutionarily more ancient (Figure 2B) would suggest that they would be more likely to have orthologs in distant species. To analyse this, and to ensure that the result is not biased by different rates of evolution, we considered a recently assembled worm-human ortholog set [33].

This set was assembled using four different orthology calling tools (InParanoid, OrthoMCL, HomoloGene and Ensembl Compara). We consider a set of worm genes with evidence for orthology in humans through any of these methods (a liberal list of 7663 genes) and a set found by all of these methods (a conservative list of 3386 genes). For each list we considered whether each member of a duplicate pair was identified as having an ortholog in humans or not. We find that duplicate genes whose double knockdown has no evident phenotype are less likely to have an ortholog in humans than duplicate genes with a knockdown phenotype (from the liberal list, 58% of non-essential genes have a human ortholog versus 84% of those with a phenotype, chi squared test, *P*<<0.0001; from the conservative list, 23% of non-essential genes have a human ortholog versus 48% of those with a phenotype, chi squared test, *P*<<0.0001). As duplicate genes without knockdown phenotype evolve faster than those with a phenotype (Figure S5), the finding of fewer genes with knockdown phenotype having an ortholog may simply reflect a higher rate of sequence evolution and hence weakened homology searching. To address this problem, we performed a logistic regression in which we predict presence or absence of orthologs in humans as a function of the knockdown phenotype and the rate of protein evolution derived from the *C. elegans*-*C. briggsae* comparison. This revealed that, while rate of protein evolution is a predictor of presence/absence of a human ortholog (liberal set: *P* = 2×10^−6^; conservative set: *P* = 4×10^−6^), duplicate genes with a double knockdown phenotype are more likely to have an ortholog in humans controlling for the rate of evolution (liberal set: *P* = 1×10^−5^; conservative set: *P* = 1×10^−7^). We conclude that duplicates genes with an underlying phenotype are more likely to be phylogenetically preserved. This result comes with the caveat that we presume the rate of evolution of a gene in the intra-worm comparison is a fair reflection of its rate of evolution in other lineages.

### Among essential genes, duplicate pairs with greater sequence similarity have higher rates of masking

**Figure 3:**
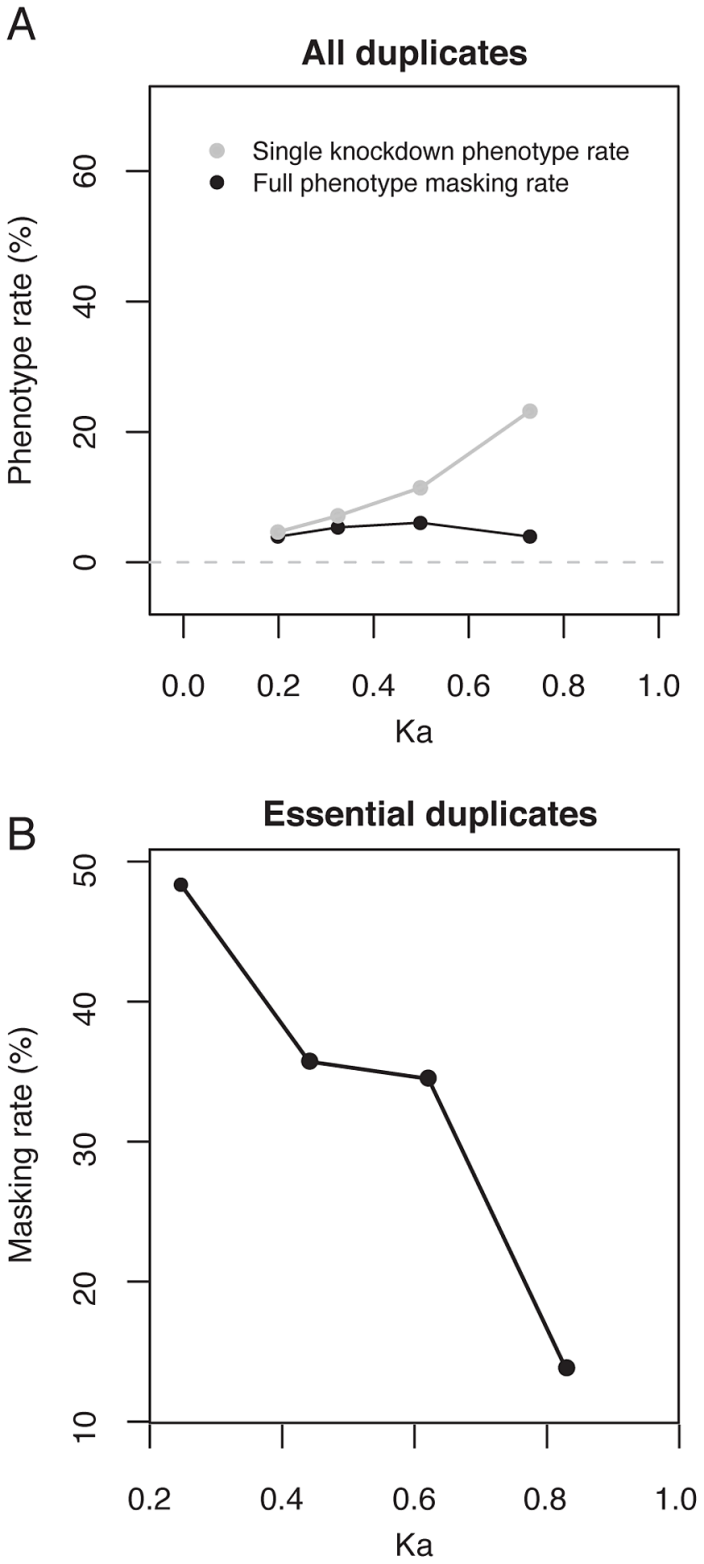
The increase in single-gene knockdown phenotype rate with Ka_pair_ is due to a retention bias for essential duplicates over duplicate age. (A) Plotted are the single-gene knockdown phenotype (grey) and fully masked phenotype (black) rates versus Ka_pair_ (protein divergence) between the two genes of a pair, for the subset of the 790 duplicate pairs for which Ka_pair_<1 (*n* = 560). For each series, datapoints are placed at the median Ka_pair_ for equivalent sized bins of duplicate genes. The single-gene knockdown phenotype rate is positively correlated with Ka_pair_ (logistic regression: *P*<10^−11^); the fully masked phenotype rate is not correlated with Ka_pair_ (logistic regression: *P* = 0.7). (B) The masking rate (full and partial) versus Ka_pair_ (protein divergence) between the two genes of a pair, for the subset of the 790 duplicate pairs for which Ka_pair_<1 and the gene-pair is essential (*n* = 115 pairs). For each series, datapoints are placed at the median Ka_pair_ for equivalent sized bins of duplicate genes. The masking rate is negatively correlated with Ka_pair_ (logistic regression: *P* = 0.002). All analyses using logistic regression were carried out on unbinned data.

It is expected that genes are most likely to exhibit masking immediately after duplication and then to show a lower rate of masking with increasing age, as they diverge in sequence and expression. This view is supported by previous studies in yeast, *C. elegans*, fly and mouse where it was observed that the single-gene knockdown phenotype rate for duplicated genes increases with protein divergence between the two members of a pair (Ka_pair_) [8], [13]–[16], [18], [24], [31] (Figure 3A; logistic regression: *P*<10^−11^). Measurement of masking rates would be made difficult by the preponderance of non-essential genes and indeed we did not find a significant correlation between Ka_pair_ and the full masking rate (Figure 3A; logistic regression: *P* = 0.7). To avoid this difficulty, we restricted analysis to essential duplicate pairs, where phenotypes are readily observed. This analysis showed a significant negative correlation between the rate of phenotype masking and Ka_pair_ (logistic regression: *P* = 0.002, Figure 3B), supporting the hypothesis that duplicate pairs with greater sequence similarity are more likely to exhibit masking. We also find a prevalence of masked phenotypes amongst the youngest duplicates (those that arose in the *C. elegans* lineage since divergence from *C. briggsae*, or in the Caenorhabditis ancestor; Figure 2C and Figure S2). Therefore, the youngest and most sequence similar duplicates are most likely to exhibit masking.

### Masking of paralogs is conserved

We were interested to test whether phenotypic masking was evolutionarily conserved. We could not compare our data to that of yeast, because only two pairs are orthologous to a yeast duplicate pair screened in yeast [24]. To assess the level of conservation of masking in a closer relative, we identified 31 duplicate pairs that arose prior to the *C. elegans-C. briggsae* speciation and tested whether the *C. briggsae* ortholog pairs showed masking (see Methods). We observed phenotype masking for 19 of the 31 *C. briggsae* duplicate pairs (61.3%), indicating significant retention of masking between duplicates over the estimated ∼30 million years [32] since the *C. elegans-C. briggsae* speciation.

### The duplication bias in favour of non-essential genes (which tend to be fast-evolving) explains in part why recently duplicated genes evolve relatively fast

Lynch and Conery [34] observed that young duplicate pairs tend to evolve fast at the protein level in *C. elegans*, mouse, human and fly and inferred that “early in their history, many gene duplicates experience a phase of relaxed selection or even accelerated evolution at replacement sites” [34]. A possible explanation for the rapid protein evolution of young duplicate pairs is that they are usually similar enough in sequence for masking to occur, and since masking compensates for mutations in either member of a duplicate pair, this may allow them to accumulate substitutions relatively rapidly [35]. The bias for successful duplication of non-essential genes suggests an alternative possibility: that this duplication bias is also a bias for successful duplication of intrinsically fast-evolving genes. This could be the case if non-essential genes evolve faster than essential genes (as some previous studies suggest [36], [37]). Indeed, when we estimated the evolutionary rate of each duplicate pair by calculating the mean protein divergence between orthologous members of the pair in *C. elegans* and *C. briggsae* (Ka_CeCb_), we find that non-essential duplicate pairs have a higher rate of protein sequence evolution than essential pairs (mean Ka_CeCb_ 0.120 vs. 0.092, Wilcoxon test: *P*<10^−4^).

Expression level is strongly negatively correlated with the rate of protein sequence evolution in many species [37], . We find that non-essential duplicate pairs have lower expression levels than essential pairs (average of 8.3-fold lower; log2 means 8.60 vs. 11.66; Wilcoxon test: *P*<10^−15^), suggesting that the higher rate of protein sequence evolution of non-essential pairs could be related to their lower expression level. In support of this, expression level is a good predictor of the rate of protein evolution (Ka_CeCb_) in an ANCOVA model (using Ln(Ka_CeCb_) as the response variable: *P*<0.0001; Figures S3 and S4). Essentiality/non-essentiality of duplicate pairs in the ANCOVA is not a significant predictor indicating that it is expression level rather than dispensability *per se* that is the important variable (Figures S3 and S4). We also find for singleton genes the difference in evolutionary rate between those with and without a phenotype on knockdown is related to differences in expression level rather than essentiality *per se* (Figures S3 and S4).

### Masking does not appear to promote rapid sequence evolution

Given these results, we propose that the relatively fast protein sequence evolution of young duplicates [34] is partly due to a bias towards successful duplication of lowly-expressed, non-essential genes, which, given their expression level, tend to evolve fast. Consistent with this, more recent duplicate pairs that arose in the Caenorhabditis ancestor have lower expression levels than duplicates that arose in the Bilaterian or Eukaryotic ancestors (average of 10.3-fold lower; log2 means 8.70 vs. 12.06; Wilcoxon test: *P*<10^−15^). Therefore, fast evolution of young/nonessential duplicates is not *prima facie* evidence that duplicates are under weak purifying selection owing to masking (as classically presumed), as young duplicates are biased towards lowly expressed non-essential genes with intrinsically high rates of evolution and, for non-essential genes, there is little or no possibility of phenotype masking. We can, however, use our data to examine this hypothesis more directly.

If duplication enabled phenotype masking and so permitted fast evolution we would expect singleton genes with an underlying phenotype to evolve slower than duplicates with an underlying phenotype. Against these expectations, for genes with a phenotype, the evolutionary rate is the same for singletons and duplicated genes (*dN* for singletons with knockdown phenotype = 0.087+/−0.094; *dN* for duplicate genes with a double knockdown phenotype = 0.092+/−0.076, t-test *P* = 0.56). Controlling for expression level does not alter this conclusion (*P* = 0.43; Figure S4). Similarly, if we compare duplicates genes with a double knockdown phenotype that show evidence of masking with those with a double knockdown phenotype but no evidence of masking we find in the ANCOVA, controlling for expression level, that presence/absence of masking is not a predictor of the rate of evolution (*P* = 0.24) (see Supplementary Result 1.1 in Text S1). Likewise singletons with a phenotype evolve no slower than duplicates with masking when controlling for expression level (P = 0.36) (see Supplementary Result 1.2 in Text S1). Incidentally, we also find that singleton genes without phenotype evolve at the same rate as duplicates genes without double knockdown phenotype (singleton genes without phenotype, dN = 0.13+/−0.1 (sd), duplicate genes without phenotype, dN = 0.12+/−0.9, t-test, P = 0.16). In sum, where there exists the possibility of phenotype masking (i.e. when the double knockdown has a phenotype), we see no evidence that the duplicated genes evolve any faster than expected of genes of similar dispensability/expression level and find no evidence that masking promotes rapid sequence evolution.

## Discussion

### Non-essential genes are more likely than essential genes to be successfully duplicated, but also to be lost in the long term

Through systematic double knockdown analyses, we showed that non-essential genes in *C. elegans* are more likely to be successfully duplicated than essential genes. A similar bias is supported by the finding of a paucity of orthologs of murine essential genes in segregating CNVs in humans [39] and the observation of lower than expected numbers of genes associated with lethal phenotypes that have copy number variants in flies [40]. The mechanism for this bias might be mutational, selectionist, or both. In a mutational model, non-essential genes could be more prone to duplication, but once duplicated no more prone to fixation than essential duplicates. Under a selectionist model, a non-essential gene could be equally prone to duplication, but the duplicate could be more likely to be fixed in the population.

Mutation bias could arise if chromosomal regions vary in their propensity for duplication, and regions with a higher density of non-essential genes have higher duplication rates. This is plausible as duplications are commonly caused by non-homologous recombination events [41], which in turn are more likely in chromosomal regions with high homologous recombination rates [42]. *C. elegans* chromosome arms have high recombination rates, are rich in duplicate genes, and are poor in essential genes [11], [43]–[45]. We hypothesise that the location of non-essential genes in chromosomal arms where the recombination rate is high might contribute to their higher propensity for duplication. Indeed, we find that 60% of younger duplicate pairs lie on the arms, compared to 30% of older pairs (Fisher test: *P* = 10^−8^), suggesting that most new duplicates arise on the arms, regions rich in non-essential genes.

The selection bias hypothesis is also plausible. In yeast, many essential genes show dosage sensitivity because they belong to protein complexes [46], [47]. Duplications of essential genes may therefore often be deleterious and purged by selection, giving rise to a net selection bias for duplications of non-essential genes. The finding that segregating CNVs in humans are depleted for orthologs of murine essential genes was interpreted in this manner [39].

As well as the bias towards duplication of non-essential genes, over the longer term we also see a retention bias for essential duplicates: essential duplicate pairs are enriched among older duplicate pairs compared to younger pairs (27.6% vs. 4.5%; Figure 2B). It is well described that in the majority of instances one of a pair of duplicates will be lost [34]. It is plausible that this death/retention process is biased, such that in the long term essential genes are more likely to persist [37]. Our data suggest that those genes that are easily duplicated (*i.e.* non-essential genes) are also more easily lost. The loss of non-essential duplicates could occur by gene loss of one of the two members (e.g. deletion, pseudogenization). Alternatively, it could be that the gene is retained but no longer recognizable as having a paralog because sequence divergence is so great. If this were the case, we would expect that essential duplicate pairs would be more slowly evolving than non-essential pairs, and as noted above, we find some evidence for this (Figure S3). However, we also find that, as noted above, the presence/absence of orthologs in humans cannot be accounted for simply in terms of differential rate of evolution; although this is a significant predictor, the presence/absence of a phenotype on knockdown also contributes significantly.

A further mechanism for loss of non-essential duplicates over time could be re-duplication of one of the members of a paralogous gene-pair. As members of a pair are defined here as each other's closest homologs, re-duplication of a non-essential duplicate gene would result in simultaneous loss of an old and creation of a young non-essential duplicate pair in our dataset. Since non-essential genes are more likely than essential genes to undergo successful duplication, they may be also more likely to undergo re-duplications.

Another possible mechanism for loss of non-essential duplicates over time could be gain of new essential functions by non-essential duplicates (*e.g. by* neofunctionalization), although experiments in yeast did not find evidence for this phenomenon [18]. Therefore, we consider that the retention bias for essential duplicate pairs is probably due to both the slower rate of divergence of essential duplicates and preferential re-duplication of non-essential duplicates.

### The rate of phenotype masking in *C. elegans* is similar to that in yeast

Genome-wide, we observed masking for 6% (50/790) of *C. elegans* duplicate pairs, roughly half that observed in the previous *C. elegans* study (11%), which was based on a smaller sample of gene-pairs (*n* = 143 [22]). This difference is probably due to a bias towards older gene-pairs in their sample compared to our genome-wide sample (Figure S6). Masking is more common among older duplicate pairs, which will have increased the observed masking rate. A masking rate of 6% for *C. elegans* paralog pairs appears to be at odds with the much higher rate of 30% observed in yeast [24]. However, our estimate for the masking rate for ‘essential’ genes, where we can confidently detect loss of function phenotypes, is 29%, very similar to the yeast estimate. Nonetheless, we note that this resemblance should be taken with the caveat of methodological differences (e.g. the yeast study used gene deletions whereas ours used RNAi knockdowns).

In conclusion, we have shown that phenotype masking makes a minor contribution to the low knockdown phenotype rate of duplicate genes. The primary reason that the knockdown phenotype rate is low is because the rate of gain and loss (or reduplication) of duplicates derived from non-essential genes is much higher than for essential genes, so that the majority of duplicate pairs are young and have arisen from non-essential precursors. While the rates of masking may differ among organisms due to the influence of varying duplication rates affecting the abundance of young non-essential duplicates, we expect the fundamental duplication dynamics presented here to be broadly applicable. In support of this, recent studies in mouse have shown that younger genes are less likely to be essential than older genes, and that there is an age dependent increase in the proportion of duplicate genes that are essential [12], [15], [16]. We conclude that phenotype masking should not be the default explanation as to why genes that have a paralog do not exhibit a discernable phenotype on single gene knockdown. It is simply more likely that they were derived from non-essential genes in the first place.

## Methods

### Identification of duplicates and RNAi clones

An all-against-all protein-sequence WU-BLAST search [48] was carried out using the longest isoform of each protein-coding gene in *C. elegans* (19735 peptides from WormBase release WS140; https://www.wormbase.org). 2690 duplicate pairs (paralog pairs) were defined as reciprocal-best matches (Table S2), requiring BLASTP matches to have an e-value less than 10^−9^ and the HSP (high-scoring pair) alignments to span a minimum of 60% of each protein. Single-copy genes were defined as proteins without a BLASTP match of e-value <0.01.

*C. elegans* RNAi bacterial reagents were obtained from Fraser *et al*, 2000 [28] and Kamath *et al*, 2003 [11]. For genes where no RNAi reagent was available, clones from the library of Rual *et al*, 2004 [30] were used. Of the 2690 pairs, 1183 pairs existed for which each gene was uniquely targeted by an RNAi reagent with no expected non-target RNAi. Unique reagents are defined in WormBase as having one primary target (gene has at least 95% nucleotide identity over 100 bp) and no predicted secondary targets (gene has at least 80% nucleotide identity over 200 bp and is not a primary target). We sequenced both clones for the 1183 pairs of RNAi reagents and found that both were correct for 932 pairs; these pairs were used for screening (Table S2).

To identify duplicate pairs without a close third paralog, we generated a measure of duplicate isolation and applied it as a filter. The ‘duplicate isolation value’ measures the protein sequence similarity between the duplicate pair relative to their similarity to the next closest BLASTP hit that they have in common (considering BLASTP hits with e-values <0.01). For comparison of relative protein-protein similarity, the negative log_10_ of BLASTP e-values was used as previously described [49]. Duplicate isolation was calculated as: negative log_10_ of the maximum e-value of the BLAST matches between the protein sequences of the duplicate pair and their closest shared hit, divided by the negative log_10_ of the e-value for the protein-sequence BLAST match between the genes of the duplicate pair (Table S2). The maximum of the e-values to the closest third paralog was used, as it should best represent the match to the third paralog from sequence shared between members of the duplicate pair. Isolation values range from 0 to 1, with 1 signifying a common best match that is equally as strong as the match between the genes of the duplicate pair, and 0 signifying that the duplicates have no match in common (*i.e.* they belong to a gene family with just two members).

To identify a subset of duplicate pairs that lack a close third paralog (but may have a distant third paralog), we filtered our set of 932 duplicate pairs using a cutoff of ≤0.83 for the duplicate isolation value. An isolation value of 0.83 would correspond, for example, to a duplicate pair with a protein-sequence BLAST match to each other of e-value 10^−100^ (or e.g. 10^−15^) and a best common BLASTP match to a third paralog of (maximum) e-value 10^−83^ (or e.g. 3.5×10^−13^). Filtering removes 142 duplicate pairs from the screened set of 932 pairs, leaving 790 duplicate pairs that lack a close third paralog, which we used for our analysis (Table S2). This threshold retains all 50 duplicate pairs that exhibited phenotype masking, indicating that the paralog pairs showing masking probably lack a third paralog that is close enough to provide masking activity.

### RNAi screen

RNAi bacteria were grown at 37°C in 96-well format in LB containing 50 µg/ml ampicillin for 6–8 hours. Cultures were concentrated 2-fold by centrifugation and removal of half of the medium before resuspension of the bacterial pellet. Aliquots of bacterial cultures targeting single genes of each paralogous pair were mixed in a 1∶1 ratio. Approximately 100 µl of each individual culture or the mixed culture was spotted onto a well of a 6-well plate containing NGM agar including 25 µg/ml carbenicillin, 1 mM IPTG and 50 µg/ml Nystatin and left to dry and induce for 36 hours.

Single and double RNAi knockdown experiments were conducted in duplicate using the RNAi hypersensitive strain *eri-1(mg366);lin-15B(n744)* [25], [26]. 5–10 L1 *eri-1;lin-15B* larvae were aliquoted per well in 40 µl drops using a WellMate liquid handling device (Matrix) from a solution of M9 buffer with 0.01% Triton X-100. Plates were incubated at 15°C for 6 days, when controls had been laying eggs for ∼24 hrs. P0s were then scored for a host of post-embryonic phenotypes (see below for F1s) before being removed by aspiration. Approximately 42 hours later, P0 fertility (Ste and Lbd) and lethality of F1 embryos (Emb) was scored. P0 mothers were scored as sterile (Ste) or low brood (Lbd) where wells contained fewer than 10 or 30 F1 progeny, respectively. Embryonic lethality (Emb) was assigned where at least 10% of the brood failed to hatch. When controls had reached mid-larval (∼66 hours) and late-larval/young adult developmental stages (∼90 hours), the F1s were scored for the following post-embryonic phenotypes: Unc (uncoordinated), Prz (paralyzed), Dpy (dumpy), Bmd (body morphology defect), Sck (sick), Bli (blister), Mlt (molting defect), Him (high incidence of males; F1s only), Pvl (protruding vulva), Muv (multivulva), Lon (long), Sma (small), Gro (growth defect), Egl (egg laying defect; P0s only), Stp (sterile progeny; F1s only), Adl (adult lethal), Ooc (oocytes laid; P0s only), Rup (ruptured), and Lvl (larval lethal). Phenotypes were assigned when at least one of the replicates had a penetrance of ≥10% in the F1 population or ≥50% for the P0 mothers. Phenotype data are given in Table S3.

Effectiveness of the double RNAi feeding was monitored by comparing single- and double knockdown phenotypes; in 99% of cases (*n* = 175) a phenotype was observed in the double feeding well when either of the single-gene knockdowns showed a phenotype. Additionally, in 92% of these cases, the double knockdown phenotype was as least as strong as that of either single knockdown indicating that the double feeding procedure was effective. Duplicate pairs showing potential phenotype masking were defined as those where double RNAi knockdown of the pair showed a stronger phenotype than either of the single-gene knockdown experiments. These candidates were retested for reproducibility; 60 confirmed pairs were subjected to a final round of quantitative testing as described below.

For 3–5 replicates, quantitative tests of brood size in the P0 generation, embryonic lethality in the F1 generation, post-embryonic lethality in the F1 generation and abnormal morphology defects in the F1 generation were carried out for single and double RNAi experiments from the progeny produced by a single P0 mother in the first 48 hours as an adult. In addition, in cases where candidates showed quantifiable phenotypes in the P0 generation, quantitative scoring of post-embryonic phenotypes was carried out for 20–30 P0s. Qualitative scoring of 20–30 P0s was also carried out, indicating the severity (e.g. severe Dpy vs. mild Dpy) or developmental stage of the phenotype (e.g. Lvl L1 vs. Lvl L4).

All quantitative phenotypes were statistically analysed to determine if the double RNAi experiment was more severe or merely mulitiplicative compared to the corresponding single RNAi experiments. Phenotype masking was defined as a genetic interaction where the double RNAi phenotype of the paralogous pair was greater than the product of each of the single-gene RNAi phenotypes, using a method adapted from Baugh *et al*, 2005 [50] as follows. Quantitative assessment of brood size, embryonic lethality, post-embryonic lethality and abnormal morphology defects were expressed as a percentage of normal development, through normalization to the same measures of 111 control animals or their progeny. The normalized phenotype is used as an estimate of the fitness of the knockdown (*w*). For each quantified phenotype, the null hypothesis was that the normalized phenotype of the double RNAi experiment (*w*_1,2_) is equal to the product of the normalized phenotypes of each of the single RNAi experiments (*w*_1_ and *w*_2_). Phenotype masking was inferred when *w*_1,2_ was significantly lower than the expected value of *w*_1_×*w*_2_ (Mann-Whitney-U test: *P*<0.05). Phenotype masking of qualitative phenotypes was inferred when either the developmental stage of the observed phenotype was earlier (e.g. Lvl L1 vs. Lvl L3), or the class of phenotype observed was more severe (e.g. Lvl L3 vs. Gro L3) in the double RNAi experiment compared to both single RNAi experiments. Following the detailed quantitative and qualitative scoring, 50 duplicate pairs were identified as showing phenotype masking (Table S4).

### Comparison to published knockdown and knockout data for *C. elegans*

We compared our RNAi phenotype data to that from genome-wide RNAi-by-feeding screens [11], [28], [29], supplemented by data from Rual *et al*, 2004 [30] where a gene lacked an RNAi reagent in the above three screens. These screens scored the same range of phenotypes as in our study. Only reagents with one primary target and no predicted secondary targets were considered (coverage for 14327 protein-coding genes). Genes targeted by an RNAi reagent that was annotated as having a loss-of-function phenotype in at least one study were assigned as having a knockdown phenotype. Of the genes having a single-gene knockdown phenotype using the combined data from Fraser-Kamath-Simmer-Rual (FKSR) screens (*n* = 198 genes), our screen also detected a single-gene knockdown phenotype in 90% of cases (Table S6). This level of concordance is similar to that observed for replicate genome-wide RNAi screens in *C. elegans* [29].

Because duplicates by nature have related sequences, we investigated the possibility that some single-gene knockdown phenotypes observed for duplicate genes that showed masking were due to RNAi off-targets (*i.e.* unintended knockdown of the other gene member of the pair) that were not predicted in WormBase. Among the set of duplicates that showed masking, we found that 100% of single genes assigned an RNAi knockdown phenotype also showed a phenotype in the genetic mutant (*n* = 19 genes, based on allele data available in WormBase). This indicates that unpredicted RNAi off-targets in the other member of a paralog pair are unlikely to have confounded estimates of phenotype masking.

### Classifying duplicate pairs as essential or non-essential

We classified each of the paralogous gene-pairs as ‘essential’ if it showed an obvious phenotypic defect upon double knockdown, or ‘non-essential’ otherwise. For this purpose, a duplicate pair was taken to have a phenotypic defect upon double knockdown if: (i) at least one gene of the pair had a (non-wildtype) phenotype based on the Fraser-Kamath-Simmer-Rual screens, or (ii) the pair showed phenotype masking in our data.

In Figure S1, as well as the gene-pairs where an RNAi probe uniquely targets each gene, we also included double knockdown phenotypes inferred for duplicate pairs that are so similar in sequence that a single RNAi reagent targets both members of the pair (*i.e.* two primary targets and no predicted secondary targets; Table S2).

### *C. briggsae* RNAi experiments

Of the 50 *C. elegans* duplicate pairs showing phenotype masking, 41 duplicate pairs were identified where the duplication giving rise to the gene-pairs occurred in the *C. elegans-C. briggsae* ancestor, resulting in two extant *C. elegans*-*C. briggsae* ortholog pairs (see Identification of orthologs below). For 31 *C. briggsae* gene-pairs, we were able to generate dsRNA to uniquely target each *C. briggsae* gene by RNAi (*i.e.* one primary target and no predicted secondary targets). Primers to amplify *C. briggsae* genomic fragments contained 5′ T7 polymerase promoter sequences (5′ TAATACGACTCACTATAGG 3′) to allow *in vitro* transcription from PCR products as described by Zipperlen *et al*, 2001 [51]. RNAi experiments were conducted with the *C. briggsae* wild-type strain (AF16). Each single dsRNA or a mixture of the two dsRNAs targeting the duplicate pair was injected into 8–10 young adult hermaphrodites at a final concentration of 1–2 µg/µl. Worms were grown at 15°C on NGM plates (2.2% agar to prevent burrowing) seeded with OP50. Injected *C. briggsae* single P0 mothers were transferred to a fresh well after 24 hours, transferred again after 48 hours and finally removed at 72 hours. Brood size and F1 progeny laid on these plates were scored beginning 24 hours after P0 transfer or final removal. Qualitative and quantitative phenotypes were scored in the same manner as described above with the exception of P0 post-embryonic scoring, given that RNAi was initiated in young adults. Quantitative data was normalized to the same measures of the P0 brood and F1 progeny of 57 *C. briggsae* worms injected with loading buffer. Following detailed quantitative and qualitative scoring, 19 duplicate pairs were identified as showing phenotype masking (Table S5).

### Estimation of the ages of duplicate gene-pairs

To estimate the dates of duplication that gave rise to duplicate pairs of *C. elegans* genes, we analysed data from the TreeFam database of animal gene families [52]. Where two genes of the pair belonged to the same TreeFam family, the duplication date was taken to be the taxonomic level of the common ancestor node for the two genes in TreeFam's phylogenetic tree for that family. Duplications were inferred to have occurred either in the *C. elegans* lineage, in the common ancestor of Caenorhabditis species, or in the common ancestor of Bilaterian species. The age estimate was considered confident if the same date was estimated from at least two of the three most recent TreeFam releases, or if there was strong support for the estimated date from the most recent TreeFam release (6). For ‘strong’ support in TreeFam 6, we required that the bootstrap for the common ancestor node was ≥70%; and that all internal nodes on the lineages back from the two genes to their common ancestor node had bootstraps of ≥70%, or were speciation nodes at which no genes had been lost. Where two genes of a duplicate pair belonged to different families, we investigated whether both families in TreeFam release 6 contained genes from human, Drosophila, and a Saccharomyces or Arabidopsis outgroup. If they did, the duplication must have occurred either in the ancestor of all eukaryotes, or in a pre-eukaryotic ancestor (e.g. the eukaryote-prokaryote common ancestor). We refer to the age of such pairs as ‘Eukaryota’. Using the above approach, we could make confident estimates for the ages of 92% of all duplicate pairs identified in *C. elegans* (*n* = 2690; Table S2).

### Identification of orthologs, and calculation of protein divergence (Ka_CeCb_) between orthologs

*C. elegans*-*C. briggsae* one-to-one orthologs were inferred from Treefam releases 4, 5 and 6, where a duplication had occurred in the *C. elegans-C. briggsae* ancestor, resulting in two extant *C. elegans*-*C. briggsae* ortholog pairs. We only retained orthologs inferred from at least 2 releases or inferred from release 6 with an orthology bootstrap of ≥70% [53], [54]. Orthologs of *C. elegans* single-copy genes in the nematodes *C. briggsae* and *C. remanei* were inferred if the orthology bootstrap was ≥70% in TreeFam release 6. Orthologs between *C. elegans* and *Saccharomyces cerevisiae* (yeast) were identified using TreeFam, and the Ensembl-Compara database [55], and found to agree in all cases examined. Protein divergence between the two members of each *C. elegans* duplicate pair (Ka_pair_, Table S2), and between *C. elegans*-*C. briggsae* orthologs (Ka_CeCb_), was measured using Li's 1993 protocol (correcting for multiple hits using Kimura's 2-parameter model) [56], [57].

### Gene Ontology analysis

Gene Ontology (GO) analysis was carried out using files obtained from the GO Consortium website http://www.geneontology.org/ (downloaded January 2012). Enrichment was assessed using Ontologizer 2.0 [58]; *P*-values were corrected for multiple hypothesis testing by Bonferroni correction.

## Supporting Information

Figure S1 Estimating the double-knockdown phenotype rate for exact duplicates. Double-knockdown phenotype rate is plotted for duplicate pairs that arose in the *C. elegans* lineage (*n* = 500) based on the ‘duplicate isolation value’, which is zero for ‘exact’ duplicates that have no other gene matches in common in the genome (i.e. no BLAST matches of e-value <0.01) and approaches one for duplicate pairs that have a close match in common to a third paralog (see Materials and Methods). Because the sample size of exact duplicates (i.e. where the gene-family size is 2; duplicate isolation value of 0) is too small for statistical testing (*n* = 28, double-knockdown rate 7.1%), we estimated the maximum double-knockdown phenotype rate for the 500 duplicate pairs that arose in the *C. elegans* lineage using the y-intercept, where no third paralog exists. This value of 12.5% is significantly less than the knockdown phenotype rate for single-copy genes (17.7%, *n* = 2566, *X* ^2^-test: *P* = 0.005; black dashed line), consistent with a bias for successful duplication of non-essential genes. The preferential duplication (and re-duplication) of non-essential genes generates less isolated duplicate pairs, consistent with the decrease in the double-knockdown phenotype rate with lower duplicate isolation. This does not exclude the possibility that buffering from a third paralog might also contribute to this trend.
(PDF)

Figure S2 Full Phenotype masking and double-knockdown phenotype rates grouped by phylogenetic age. (A) Fully masked phenotype rates (i.e. if no observable defect is found upon single-gene knockdown, but phenotype masking is revealed upon double gene knockdown) for the subset of the 790 duplicate pairs (without a close third paralog) for which phylogenetic age could be estimated (*n* = 711 pairs for whole set; *C. elegans n* = 178; Caenorhabditis *n* = 442; Bilateria *n* = 57; Eukaryota *n* = 34). (B) Double-knockdown phenotype rate for duplicate pairs in (A). (C) Fully masked phenotype rates for duplicate pairs in (A) considering only duplicates with a double-knockdown phenotype (*n* = 155 pairs for whole set; *C. elegans n* = 8; Caenorhabditis *n* = 92; Bilateria *n* = 26; Eukaryota *n* = 29). Fully masked phenotype rates differ according to phylogenetic age (Fisher's test: *P*<10^−6^), with a prevalence of full masking amongst younger duplicate pairs. (D) Number of pairs analysed for duplicate pairs in (A).
(PDF)

Figure S3 Analysis of rates of evolution of genes by effects of knock-downs I. Rates of protein evolution were calculated using the method of Li, 1993 [56] by comparing a *C. elegans* gene to its *C. briggsae* ortholog. The WormBase (WS233) defined ortholog set was employed. As some rates of protein evolution were zero we added one to the *dN* and took the natural log. Expression level is taken from *C. elegans* microarray expression data of [59]. Genes without knockdown phenotypes are represented as squares: red = duplicate genes without phenotypic effects on double knockdown (*dN* and expression rate are the mean for the orthologous pair of genes); grey = singleton genes without phenotype on single gene knockdown. Singleton here refers to the gene's status in *C. elegans*. In circles are genes with phenotypes on knockdown: blue for duplicate genes with double knockdown phenotype; green for singleton genes with phenotypes. The red and blue lines are the ANCOVA lines for the duplicate genes comparing those with and without phenotype. Expression level is the covariate and evolutionary rate is the response variable. Note that while duplicate genes with and without phenotype have different mean rates of evolution, this is because they are expressed at different levels (hence the blue and red ANCOVA regression lines intercept the Y axis at almost the same point). Presence/absence of a phenotype is not a predictor in the ANCOVA (*P* = 0.3). Comparing singleton genes we find that singletons without a phenotype evolve faster than those with a phenotype (P = 6×10^−7^), but this is owing to their being expressed at different levels. In the ANCOVA for the singletons, the interaction term is not significant (permitting ANCOVA to be performed). In this ANCOVA the effect of phenotype is not significant (*P* = 0.09) while expression level is highly significant (P<10^−12^). ANCOVA lines comparing singleton genes with and without phenotype are shown in green and grey. Singleton genes without phenotype evolve at the same rate as duplicate genes without double knockdown phenotype (singleton genes without phenotype, dN = 0.13+/−0.1 (sd), duplicate genes without phenotype, dN = 0.12+/−0.9, t test, P = 0.16 (also robust to non-parametric test). Similarly, for genes with a phenotype, the evolutionary rate is the same for singletons and duplicate genes (dN for singletons with knockdown phenotype = 0.087+/−0.094; dN for duplicate genes with a double knockdown phenotype = 0.092+/−0.076, t-test *P* = 0.56 (also robust to non-parametric test)). For both singletons and duplicates those with a phenotype are expressed at higher levels than those without (for singletons, P<2×10^−16^; for duplicates, P<2×10^−16^). Singletons are generally more highly expressed than the comparable set of genes that have a duplicate: singletons without phenotype versus duplicate genes without a double knockdown phenotype (P<2×10^−16^); singletons with a phenotype versus duplicates with a knockdown phenotype (P<3×10^−11^). Thus duplicate genes without a phenotype are the least expressed and singletons with a phenotype are the most highly expressed. These results are robust to the use of RNA-Seq based expression data (Figure S4).
(PDF)

Figure S4 Analysis of rates of evolution of genes by effects of knock-downs II. We repeated the analysis shown in Figure S3, using more extensive RNA-Seq based expression data [60]. The rate of protein evolution was calculated as described in Figure S3. In square are genes without phenotype on knockdown: red = duplicate genes without phenotypic effects on double knockdown (dN and expression rate are the mean for the orthologous pair of genes); grey = singleton genes without phenotype on single gene knockdown. In circles are genes with phenotypes on knockdown: blue for duplicate genes with double knockdown phenotype; green for singleton genes with phenotypes. As with the microarray expression data set used in Figure S3, the duplicate genes without a phenotype have lower expression levels than genes with a double knockdown phenotype (P = 3×10^−8^). With expression level as a key predictor of rates of protein evolution it is again vital to control for this variable via an ANCOVA. The red and blue lines are the ANCOVA lines for the duplicate genes comparing those with and without phenotype. As before, while duplicate genes with and without phenotype have different mean rates of evolution, this is because they are expressed at different levels (hence the ANCOVA regression lines intercept the Y axis at almost the same point). Presence/absence of a phenotype is not a predictor in the ANCOVA (*P* = 0.33). Expression level remains the only predictor of rates of evolution in the duplicate gene set (P<10^−15^). Again we find that singletons without a phenotype evolve faster than those with a phenotype owing to their being expressed at different levels. In the ANCOVA for the singletons, the interaction term is not significant (permitting ANCOVA to be performed). In this ANCOVA the effect of phenotype is not significant (*P* = 0.8) while expression level is highly significant (P<10^−24^). ANCOVA lines comparing singleton genes with and without phenotype are shown in green and grey. (but are so close that only one is readily visible). For both singletons and duplicates those with a phenotype are expressed at higher levels than those without (for singletons, P<2×10^−12^; for duplicates, P<2×10^−18^). Singletons are generally more highly expressed than the comparable set of genes that have a duplicate: singletons without phenotype versus duplicate genes without a double knockdown phenotype (P<08×10^−10^); singletons with a phenotype versus duplicates with a knockdown phenotype (P<0.0007). Thus duplicate genes without a phenotype are the least expressed and singletons with a phenotype are the most highly expressed. Do singleton genes without a phenotype and duplicate genes without a phenotype evolve at the same rate controlling for expression level? Similarly, do singleton genes with a phenotype and duplicate genes with a phenotype evolve at the same rate controlling for expression level? To estimate this we considered a regression of all data with the log of expression level predicting the log (evolutionary rate +1). We then examine the residuals of this plot, thus controlling for expression level. Doing this we find that singleton genes without a phenotype evolve faster than duplicate genes without a double knock-down phenotype, when the difference in expression level is controlled (*P* = 1.5×10^−6^). This may reflect a duplication bias favouring intrinsically fast evolving genes. However, where there is an underlying phenotype singletons and duplicates evolve at the same rate controlling for expression level (*P* = 0.43).
(PDF)

Figure S5 Protein divergence rates of duplicate genes. Protein divergence (Ka) between *C. elegans*-*C. briggsae* one-to-one orthologs, where a duplication had occurred in the *C. elegans-C. briggsae* ancestor, resulting in two extant *C. elegans*-*C. briggsae* ortholog pairs. Genes that are members of *C. elegans* duplicate pairs that show a double-knockdown phenotype (Essential; *n* = 272) were found to have significantly lower divergence with respect to their *C. briggsae* orthologs, compared to members of *C. elegans* duplicate pairs that do not show any double-knockdown phenotype (Non-essential; *n* = 544; Means 0.094 vs. 0.123; Mann-Whitney-U test: *P*<10^−8^).
(PDF)

Figure S6 Comparison of phylogenetic age distributions of *C. elegans* duplicate pairs tested in this study and by Tischler *et al*, 2006 [22]. Phylogenetic age could be assigned for 711 of 790 duplicate pairs without a close third paralog (black) (*C. elegans n* = 178; Caenorhabditis *n* = 442; Bilateria *n* = 57; Eukaryota *n* = 34) in our data set and for 132 of 143 duplicate pairs tested in Tischler *et al*, 2006 [22] (*C. elegans n* = 9; Caenorhabditis *n* = 115; Bilateria *n* = 7; Eukaryota *n* = 1). The Tischler *et al*, 2006 [22] study contains a significantly greater proportion of older paralogs, which arose from duplications in the Caenorhabditis ancestor or earlier, compared to this study (93% vs. 75%; *X* ^2^-test: *P*<10^−5^). After controlling for evolutionary conservation and duplicate age by considering only essential duplicates (since essential duplicates are more slowly evolving) that arose in the Caenorhabditis ancestor, we found no significant difference between the masking rates in this study (*n* = 92, 40%) and Tischler *et al*, 2006 [22] (*n* = 27, 44%; *X* ^2^-test: *P* = 0.9).
(PDF)

Table S1 Distribution and enrichment of Gene Ontology (GO) functional terms among the 178 younger *C. elegans* duplicate gene pairs compared to (a) the genome, and (b) the set of all duplicate gene pairs tested for masking.
(XLS)

Table S2 2690 *C. elegans* duplicate pairs as described in Materials and Methods.
(XLS)

Table S3 Single and double RNAi phenotypes for the 790 *C. elegans* duplicate pairs screened in this study.
(XLS)

Table S4 Summary of masked phenotypes for the 50 *C. elegans* duplicate pairs that exhibited masking.
(XLS)

Table S5 Summary of masked phenotypes in *C. briggsae*.
(XLS)

Table S6 Single and double RNAi phenotypes for the 790 *C. elegans* duplicate pairs observed in this study compared to single RNAi phenotypes.
(XLS)

Text S1 No evidence that masked genes evolve fast. Supplementary Result 1.1: Masked genes evolve no faster than unmasked genes with an underlying phenotype. Supplementary Result 1.2: Masked genes evolve no faster than singleton genes with an underlying phenotype.
(DOCX)
