# Supplementary figures and images for: Duplication and Retention Biases of Essential and Non-Essential Genes Revealed by Systematic Knockdown Analyses
Source: PLoS Genet. 2013 May 9;9(5):e1003330. doi: 10.1371/journal.pgen.1003330 (PMC3649981; doi:10.1371/journal.pgen.1003330)

Supplementary Figure 1

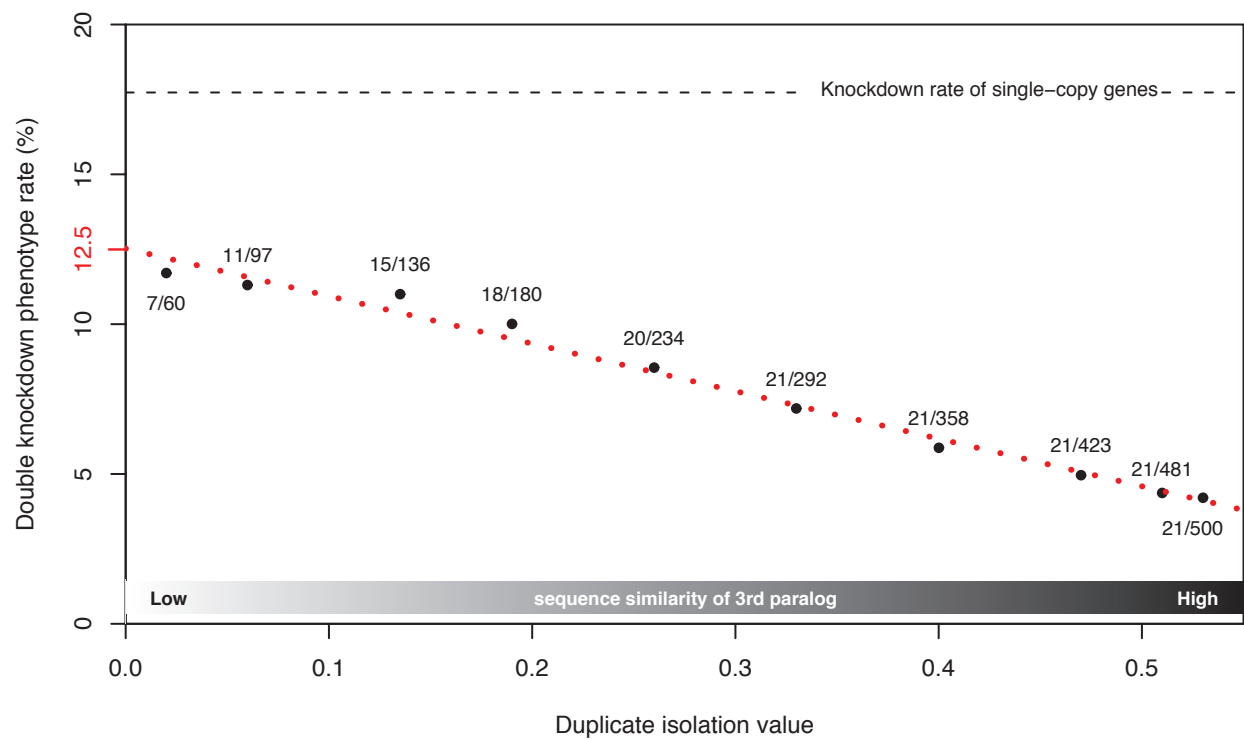

Supplement: Figure S1 — Estimating the double-knockdown phenotype rate for exact duplicates. Double-knockdown phenotype rate is plotted for duplicate pairs that arose in the C. elegans lineage (n = 500) based on the ‘duplicate isolation value’, which is zero for ‘exact’ duplicates that have no other gene matches in common in the genome (i.e. no BLAST matches of e-value <0.01) and approaches one for duplicate pairs that have a close match in common to a third paralog (see Materials and Methods). Because the sample size of exact duplicates (i.e. where the gene-family size is 2; duplicate isolation value of 0) is too small for statistical testing (n = 28, double-knockdown rate 7.1%), we estimated the maximum double-knockdown phenotype rate for the 500 duplicate pairs that arose in the C. elegans lineage using the y-intercept, where no third paralog exists. This value of 12.5% is significantly less than the knockdown phenotype rate for single-copy genes (17.7%, n = 2566, X 2-test: P = 0.005; black dashed line), consistent with a bias for successful duplication of non-essential genes. The preferential duplication (and re-duplication) of non-essential genes generates less isolated duplicate pairs, consistent with the decrease in the double-knockdown phenotype rate with lower duplicate isolation. This does not exclude the possibility that buffering from a third paralog might also contribute to this trend. (PDF) [file pgen.1003330.s001.pdf]

Supplementary Figure 2

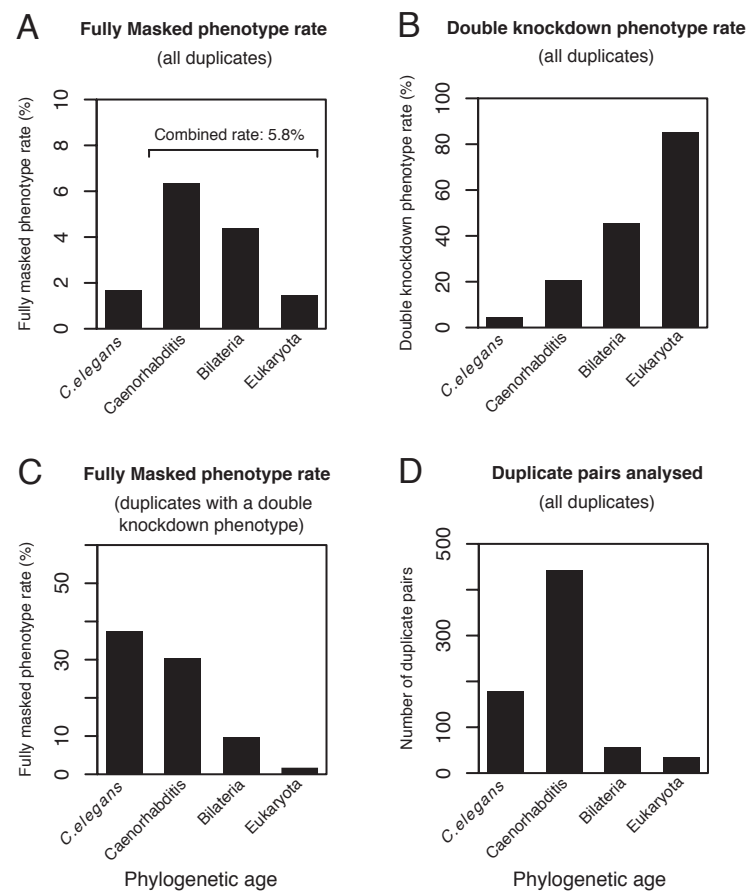

Supplement: Figure S2 — Full Phenotype masking and double-knockdown phenotype rates grouped by phylogenetic age. (A) Fully masked phenotype rates (i.e. if no observable defect is found upon single-gene knockdown, but phenotype masking is revealed upon double gene knockdown) for the subset of the 790 duplicate pairs (without a close third paralog) for which phylogenetic age could be estimated (n = 711 pairs for whole set; C. elegans n = 178; Caenorhabditis n = 442; Bilateria n = 57; Eukaryota n = 34). (B) Double-knockdown phenotype rate for duplicate pairs in (A). (C) Fully masked phenotype rates for duplicate pairs in (A) considering only duplicates with a double-knockdown phenotype (n = 155 pairs for whole set; C. elegans n = 8; Caenorhabditis n = 92; Bilateria n = 26; Eukaryota n = 29). Fully masked phenotype rates differ according to phylogenetic age (Fisher's test: P<10−6), with a prevalence of full masking amongst younger duplicate pairs. (D) Number of pairs analysed for duplicate pairs in (A). (PDF) [file pgen.1003330.s002.pdf]

Figure S3

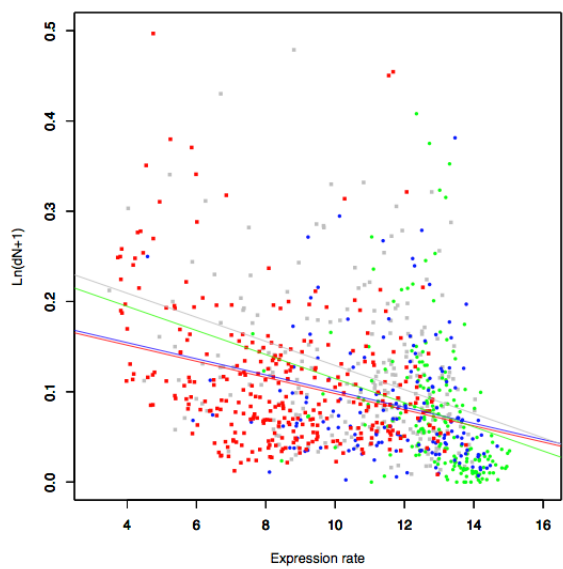

Supplement: Figure S3 — Analysis of rates of evolution of genes by effects of knock-downs I. Rates of protein evolution were calculated using the method of Li, 1993 [56] by comparing a C. elegans gene to its C. briggsae ortholog. The WormBase (WS233) defined ortholog set was employed. As some rates of protein evolution were zero we added one to the dN and took the natural log. Expression level is taken from C. elegans microarray expression data of [59]. Genes without knockdown phenotypes are represented as squares: red = duplicate genes without phenotypic effects on double knockdown (dN and expression rate are the mean for the orthologous pair of genes); grey = singleton genes without phenotype on single gene knockdown. Singleton here refers to the gene's status in C. elegans. In circles are genes with phenotypes on knockdown: blue for duplicate genes with double knockdown phenotype; green for singleton genes with phenotypes. The red and blue lines are the ANCOVA lines for the duplicate genes comparing those with and without phenotype. Expression level is the covariate and evolutionary rate is the response variable. Note that while duplicate genes with and without phenotype have different mean rates of evolution, this is because they are expressed at different levels (hence the blue and red ANCOVA regression lines intercept the Y axis at almost the same point). Presence/absence of a phenotype is not a predictor in the ANCOVA (P = 0.3). Comparing singleton genes we find that singletons without a phenotype evolve faster than those with a phenotype (P = 6×10^−7^), but this is owing to their being expressed at different levels. In the ANCOVA for the singletons, the interaction term is not significant (permitting ANCOVA to be performed). In this ANCOVA the effect of phenotype is not significant (P = 0.09) while expression level is highly significant (P<10−12). ANCOVA lines comparing singleton genes with and without phenotype are shown in green and grey. Singleton genes without phenotype evolve at [file pgen.1003330.s003.pdf]

Figure S4

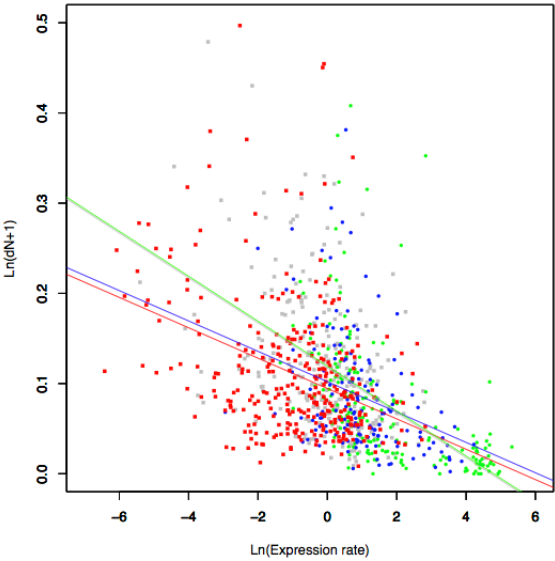

Supplement: Figure S4 — Analysis of rates of evolution of genes by effects of knock-downs II. We repeated the analysis shown in Figure S3, using more extensive RNA-Seq based expression data [60]. The rate of protein evolution was calculated as described in Figure S3. In square are genes without phenotype on knockdown: red = duplicate genes without phenotypic effects on double knockdown (dN and expression rate are the mean for the orthologous pair of genes); grey = singleton genes without phenotype on single gene knockdown. In circles are genes with phenotypes on knockdown: blue for duplicate genes with double knockdown phenotype; green for singleton genes with phenotypes. As with the microarray expression data set used in Figure S3, the duplicate genes without a phenotype have lower expression levels than genes with a double knockdown phenotype (P = 3×10^−8^). With expression level as a key predictor of rates of protein evolution it is again vital to control for this variable via an ANCOVA. The red and blue lines are the ANCOVA lines for the duplicate genes comparing those with and without phenotype. As before, while duplicate genes with and without phenotype have different mean rates of evolution, this is because they are expressed at different levels (hence the ANCOVA regression lines intercept the Y axis at almost the same point). Presence/absence of a phenotype is not a predictor in the ANCOVA (P = 0.33). Expression level remains the only predictor of rates of evolution in the duplicate gene set (P<10−15). Again we find that singletons without a phenotype evolve faster than those with a phenotype owing to their being expressed at different levels. In the ANCOVA for the singletons, the interaction term is not significant (permitting ANCOVA to be performed). In this ANCOVA the effect of phenotype is not significant (P = 0.8) while expression level is highly significant (P<10−24). ANCOVA lines comparing singleton genes with and without phenotype are shown in green and grey. (but are so clos [file pgen.1003330.s004.pdf]

Figure S5

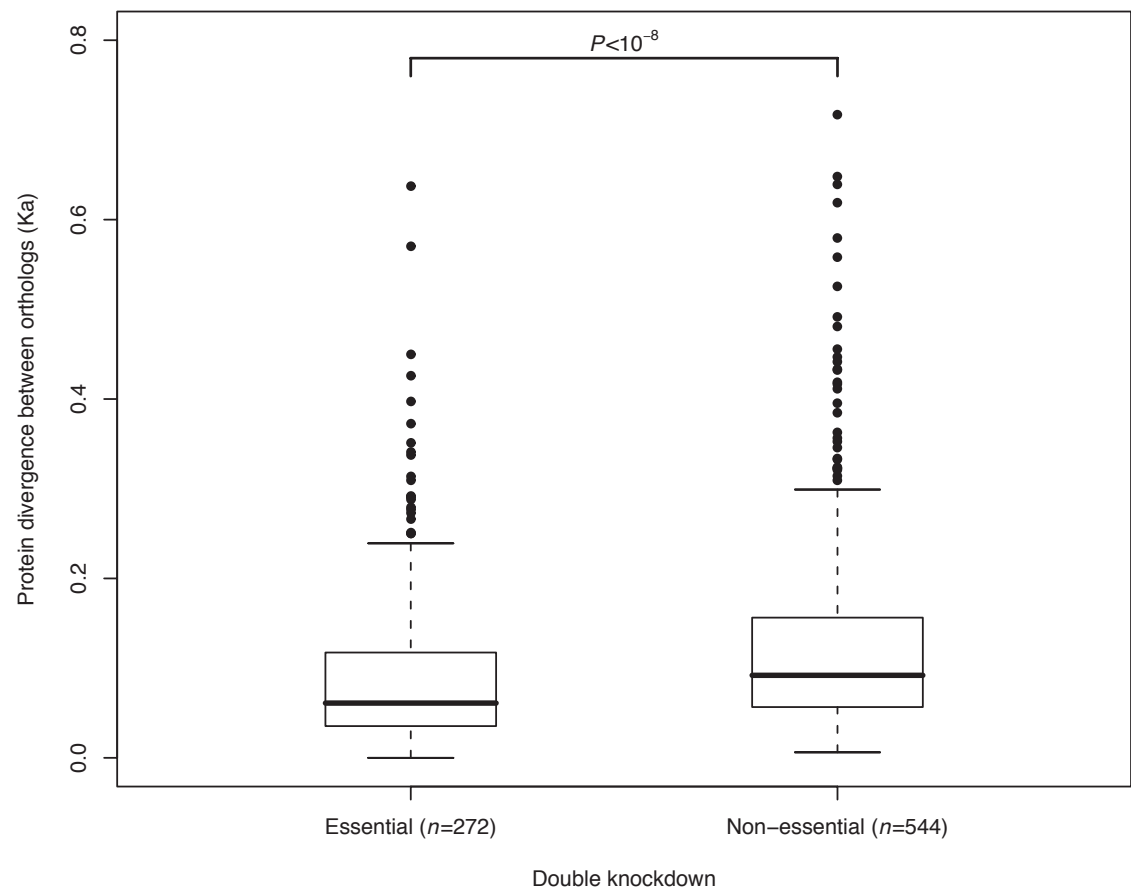

Supplement: Figure S5 — Protein divergence rates of duplicate genes. Protein divergence (Ka) between C. elegans-C. briggsae one-to-one orthologs, where a duplication had occurred in the C. elegans-C. briggsae ancestor, resulting in two extant C. elegans-C. briggsae ortholog pairs. Genes that are members of C. elegans duplicate pairs that show a double-knockdown phenotype (Essential; n = 272) were found to have significantly lower divergence with respect to their C. briggsae orthologs, compared to members of C. elegans duplicate pairs that do not show any double-knockdown phenotype (Non-essential; n = 544; Means 0.094 vs. 0.123; Mann-Whitney-U test: P<10−8). (PDF) [file pgen.1003330.s005.pdf]

Figure S6

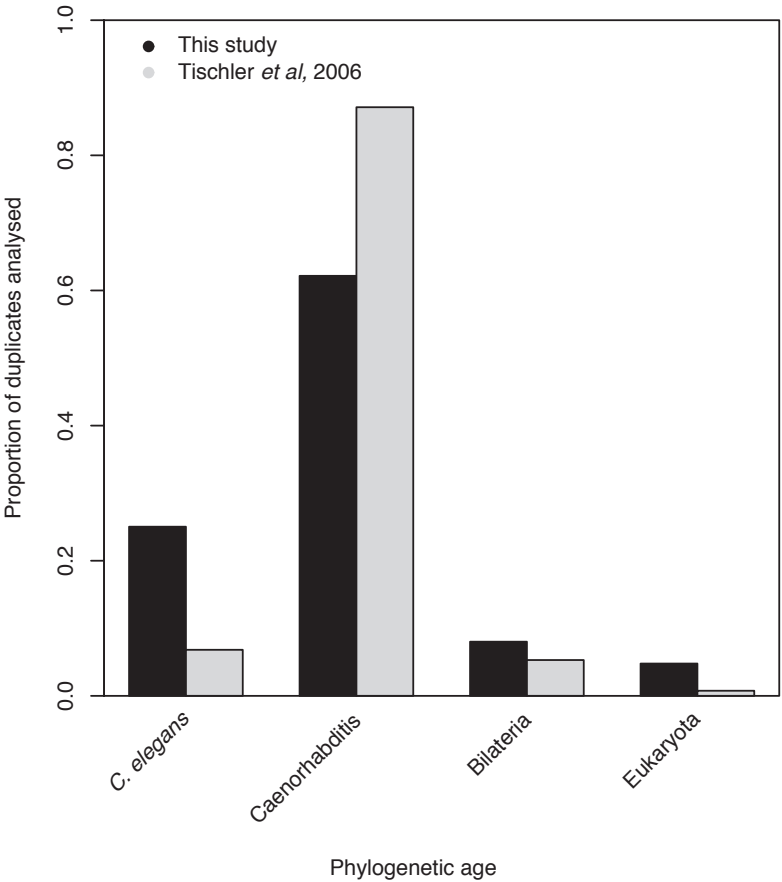

Supplement: Figure S6 — Comparison of phylogenetic age distributions of C. elegans duplicate pairs tested in this study and by Tischler et al, 2006 [22]. Phylogenetic age could be assigned for 711 of 790 duplicate pairs without a close third paralog (black) (C. elegans n = 178; Caenorhabditis n = 442; Bilateria n = 57; Eukaryota n = 34) in our data set and for 132 of 143 duplicate pairs tested in Tischler et al, 2006 [22] (C. elegans n = 9; Caenorhabditis n = 115; Bilateria n = 7; Eukaryota n = 1). The Tischler et al, 2006 [22] study contains a significantly greater proportion of older paralogs, which arose from duplications in the Caenorhabditis ancestor or earlier, compared to this study (93% vs. 75%; X 2-test: P<10−5). After controlling for evolutionary conservation and duplicate age by considering only essential duplicates (since essential duplicates are more slowly evolving) that arose in the Caenorhabditis ancestor, we found no significant difference between the masking rates in this study (n = 92, 40%) and Tischler et al, 2006 [22] (n = 27, 44%; X 2-test: P = 0.9). (PDF) [file pgen.1003330.s006.pdf]
