# Supplementary material for: Duplication and Retention Biases of Essential and Non-Essential Genes Revealed by Systematic Knockdown Analyses
Source: PLoS Genet. 2013 May 9;9(5):e1003330. doi: 10.1371/journal.pgen.1003330 (PMC3649981; doi:10.1371/journal.pgen.1003330)
Supplement: Text S1 — No evidence that masked genes evolve fast. Supplementary Result 1.1: Masked genes evolve no faster than unmasked genes with an underlying phenotype. Supplementary Result 1.2: Masked genes evolve no faster than singleton genes with an underlying phenotype. (DOCX) [file pgen.1003330.s013.docx]

**Text S1. No evidence that masked genes evolve fast.**

It is often conjectured that masking of one paralog by another can enable otherwise deleterious mutations to be rendered neutral, hence permitting rapid evolution of masked genes. This has been suggested to explain why young duplicates evolve relatively fast. We have shown that a major component of the fast evolution of young duplicates is that they are biased to intrinsically fast evolving genes with low expression levels. Is there then any evidence that masking *per se* also accelerates evolution? To address this we performed two ANCOVAs in which we control for expression level. First we compared duplicates that show masking and those that do not show masking but do have an underlying phenotype (so could have provided evidence for masking). Second we compared the set of masked duplicates against singletons with an underlying phenotype. For expression level, we used RNAseq expression data of [[60](#_ENREF_60)] and only considered genes for which there is expression data. Rates of protein evolution were calculated using the method of Li, 1993 [[56](#_ENREF_56)] by comparing a *C. elegans* gene to its *C. briggsae* ortholog. The WormBase (WS233) defined ortholog set was employed. As some rates of protein evolution were zero we added one to the dN and took the natural log.

**Supplementary Result 1.1 Masked genes evolve no faster than unmasked genes with an underlying phenotype.**

In an uncontrolled analysis masked genes evolve no faster than unmasked duplicates with an underlying double knockdown phenotype (dN for masked =0.11+/-0.017, sem; dN for unmasked = 0.097 +/- 0.01; Mann Whitney U test P=0.15). The sets do not significantly differ in expression level (P=0.77). Nonetheless, to be cautious we consider the ANCOVA comparing the two groups with expression as a covariate. We employ Ln(expression rate) and Ln(dN+1) in the analysis. The interaction term is not significant (P=0.848) so the ANCOVA is legitimate. In the ANCOVA, expression is a highly significant predictor of the rate of protein evolution (p=1.9 x 10^-7^) but whether or not the duplicate is masked is not a predictor (P=0.24). We conclude that masked and unmasked duplicates with an underlying phenotype on double knockdown do not evolve at different rates.

**Supplementary Result 1.2 Masked genes evolve no faster than singleton genes with an underlying phenotype.**

In an uncontrolled analysis masked genes evolve faster than singletons genes with a knockdown phenotype (dN for masked =0.11+/-0.017, sem; dN for singletons with phenotype= 0.081 +/- 0.006*; Mann Whitney U test P=0.03). However, the two sets differ significantly in expression level (*P*=0.03) with the singletons having higher expression levels. It is thus important that we consider the ANCOVA comparing the two groups with expression as a covariate. We employ Ln(expression rate) and Ln(dN+1) in the analysis. The interaction term is not significant (P=0.64) so the ANCOVA is legitimate. In the ANCOVA, expression is a highly significant predictor of the rate of protein evolution (P=3 x 10^-14^) but whether or not the duplicate is masked is not a predictor (P=0.36). We conclude that masked duplicates and singleton genes with an underlying phenotype on double knockdown do not evolve at different rates when controlling for expression level.

**Summary:** Both analysis find no evidence that, controlling for expression level, masked genes evolve any differently from any other gene with an underlying phenotype on knockdown.

*Note this figure is slightly lower than that given in the main text dN for singletons (dN= 0.087) as here we consider only those genes for which expression data is available.
